# Acetylacetone Photolysis at 280 nm Studied by Velocity-Map Ion Imaging

**DOI:** 10.1021/acs.jpca.3c01653

**Published:** 2023-08-03

**Authors:** Johanna E. Rinaman, Craig Murray

**Affiliations:** Department of Chemistry, University of California, Irvine, Irvine, California 92697, United States

## Abstract

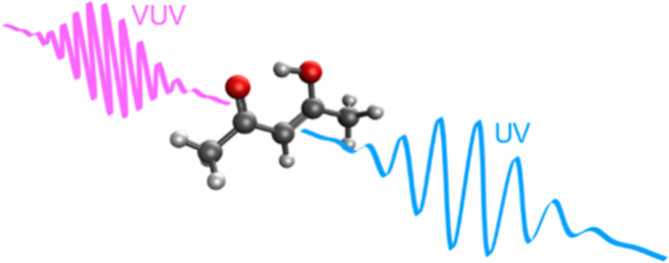

The photolysis of acetylacetone (AcAc) has been studied using velocity-map ion imaging with pulsed nanosecond lasers. The enolone tautomer of AcAc (CH_3_C(O)CH=C(OH)CH_3_) was excited in the strong UV absorption band by UV pulses at 280 nm, preparing the S_2_(ππ*) state, and products were probed after a short time delay by single-photon VUV ionization at 118.2 nm. Two-color UV + VUV time-of-flight mass spectra show enhancement of fragments at *m*/*z* = 15, 42, 43, 58, and 85 at the lowest UV pulse energies and depletion of the parent ion at *m*/*z* = 100. Ion images of the five major fragments are all isotropic, indicating dissociation lifetimes that are long on the timescale of molecular rotation but shorter than the laser pulse duration (<6 ns). The *m*/*z* = 15 and 85 fragments have identical momentum distributions with moderate translational energy release, suggesting that they are formed as a neutral product pair and likely via a Norrish type I dissociation of the enolone to form CH_3_ + C(O)CH=C(OH)CH_3_ over a barrier on a triplet surface. The *m*/*z* = 43 fragment may be tentatively assigned to the alternative Norrish type I pathway that produces CH_3_CO + CH_2_C(O)CH_3_ on S_0_ following phototautomerization to the diketone, although alternative mechanisms involving dissociative ionization of a larger primary photoproduct cannot be conclusively ruled out. The *m*/*z* = 42 and 58 fragments are not momentum-matched and consequently are not formed as a neutral pair via a unimolecular dissociation pathway on S_0_. They also likely originate from the dissociative ionization of primary photofragments. RRKM calculations suggest that unimolecular dissociation pathways that lead to molecular products on S_0_ are generally slow, implying an upper-limit lifetime of <46 ns after excitation at 280 nm. Time-dependent measurements suggest that the observed photofragments likely do not arise from dissociative ionization of energized AcAc S_0_*.

## Introduction

Interest in the atmospheric fate and possible environmental impact of acetylacetone (AcAc, 2,4-pentanedione) has been driven in part by its wide use in industry.^1−3^ Degradation of AcAc in the troposphere is dominated by photolysis, while reactions with atmospheric oxidants contribute to a much lesser extent.^1,4−6^ The tropospheric lifetime of AcAc due to reaction with OH radicals has been estimated to be around 4 h while that for photolysis is only 45 min.^1,5,6^ Consequently, understanding the photochemistry of AcAc is important to assess its potential role in tropospheric chemistry.

**Scheme 1:**
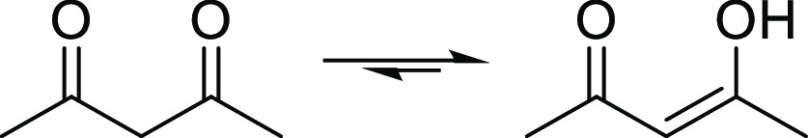
Keto-enol Tautomerization in AcAc

AcAc exists as both diketone [CH_3_C(O)CH_2_C(O)CH_3_, 2,4-pentanedione] and enolone [CH_3_C(O)CH=C(OH)CH_3_, 4-hydroxypent-3-en-2-one] tautomers in the gas phase (shown in Scheme 1 and henceforth labeled as keto-AcAc and enol-AcAc), with the equilibrium strongly favoring the latter near room temperature (∼96%).^7−10^

**Figure 1:**
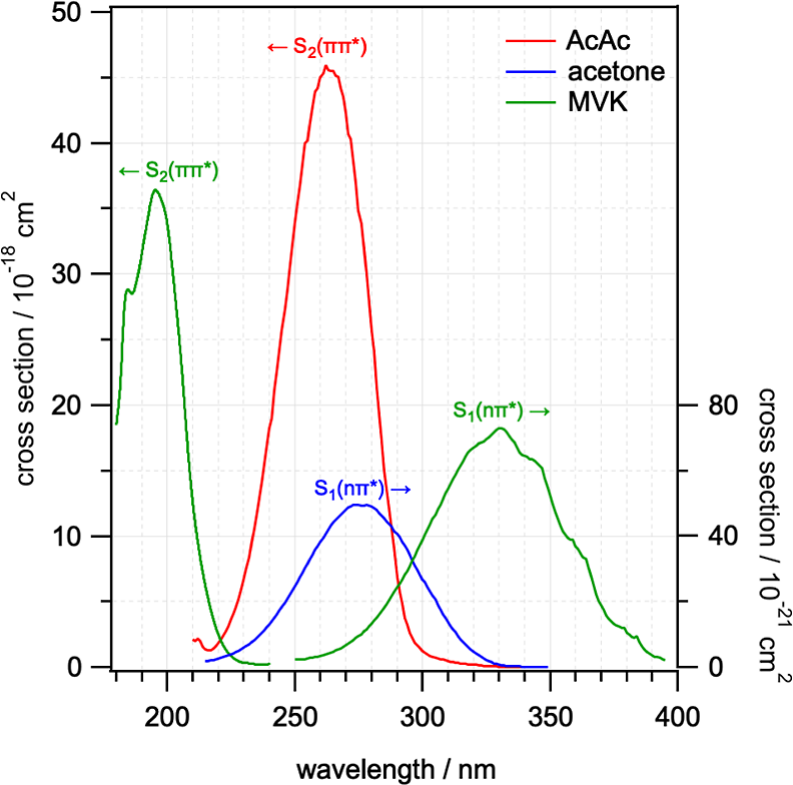
UV absorption spectra for acetylacetone (AcAc), acetone, and MVK. Data obtained from various sources.^6,11−13^

The UV absorption spectra of AcAc and other carbonyl-containing species are shown in Figure 1.^6,11−13^ The AcAc spectrum is dominated by an intense structureless band centered at 263 nm, accompanied by a much weaker feature at ∼295 nm. The strong UV absorption of AcAc has been attributed to excitation of the S_2_(ππ*) state of enol-AcAc, with the weaker feature attributed to the S_1_(nπ*) state of keto-AcAc.^6,8^ Electron-impact spectroscopy measurements have also identified a weak spin-forbidden band at 347 nm.^14^ The peak cross section is almost 3 orders of magnitude larger than the typical S_1_(nπ*) absorption of simple monocarbonyls such as acetone (also shown in Figure 1). As the spectrum is dominated by the enolone conformer, a more appropriate comparison is to α,β-unsaturated carbonyls such as acrolein or methyl vinyl ketone (MVK, also shown in Figure 1), which share the same C=C–C=O moiety. The transition to the S_2_(ππ*) state in enol-AcAc is much more red-shifted than in MVK, which has been ascribed to the aromatic character of the six-membered ring structure.^15^

The early time dynamics of AcAc following excitation to S_2_(ππ*) have been probed by several groups using ultrafast electron diffraction, photoelectron/photoion spectroscopy, and transient X-ray absorption spectroscopy.^16−20^ According to the more recent work, S_2_(ππ*) → S_1_(nπ*) internal conversion (IC) occurs on an ultrafast timescale (<100 fs).^18−20^ Rapid intersystem crossing (ISC) then transfers the population from S_1_(nπ*) → T_1_(ππ*) with a time constant of ∼1.5 ps,^18,20^ although this step has also been assigned to S_2_ → S_1_ IC.^17^ Ab initio calculations have suggested that the S_1_ → T_1_ ISC step may be facilitated by passage through a triple conical intersection between S_1_ and the low-lying T_2_(nπ*) and T_1_(ππ*) states.^19,21^ However, the precise role of the T_2_ state in the dynamics, if any, is not clear as the S_1_ → T_1_ ISC would also be strongly favored by El-Sayed’s rules.^22^ Kotsina et al*.* have assigned time constants of 20 and 330 ps to relaxation of the T_1_ state and T_1_ → S_0_ ISC, respectively.^20^ Collectively, these non-radiative processes efficiently return the population to S_0_ on a sub-nanosecond timescale. The initial non-radiative pathways exhibit similar timescales to those observed in a series of simple α,β-unsaturated carbonyls (acrolein, crotonaldehyde, methacrolein, and MVK) following excitation to the S_2_ state, where S_2_ → S_1_ IC takes place on a <200 fs timescale and subsequent S_1_ → S_0_ IC occurs on a 360–2020 fs timescale, with a marked dependence on the substituents.^23^ ISC to triplet states appears not to be competitive, although there is evidence from the photolysis of acrolein following excitation to either the S_2_ or S_1_ state that a minor channel forming HCO + C_2_H_3_ occurs non-statistically over a barrier on a triplet surface.^24,25^ Overall, photolysis quantum yields appear to be small for α,β-unsaturated carbonyls.^26^

The most comprehensive characterization of AcAc photodissociation comes from Antonov et al.,^10^ who used a combination of photoionization mass spectrometry (PIMS), photoelectron-photoion coincidence (PEPICO) spectroscopy, and IR laser absorption experiments, alongside supporting ab initio calculations. Six distinct one-photon channels were identified following excitation at 248 nm (5.00 eV) and 266 nm (4.66 eV),^10^ most originating on the S_0_ surface. The major pathway was found to be phototautomerization to keto-AcAc on S_0_, with a product yield of 29%, in agreement with earlier work in cryogenic matrices.^27−29^ The major one-photon dissociation channels formed methane + acetyl ketene and water + acetyl allene, with product yields of 12 and 25%, respectively. The former channel was attributed to dissociation over a relatively low-energy transition state (TS) on S_0_ identified by the ab initio calculations. A TS for the latter channel was also identified but at a significantly higher energy. Due to the relatively large yield, it was proposed that dissociation occurred on an electronically excited triplet surface. Two minor channels involved the formation of ketene with both acetone and propen-2-ol co-products resulting from the dissociation of both AcAc tautomers via accessible TSs on the S_0_ surface. The only radical channel observed was the Norrish type I products acetyl + acetonyl (Φ = 0.05), which was attributed to barrierless dissociation of keto-AcAc on S_0_. All other products observed were attributed to two-photon dissociation mechanisms, including the hydroxyl radical OH that had previously been thought to be a primary product resulting from one-photon dissociation.^10,30−32^ However, ab initio calculations at the CBS-QB3 level found that formation of OH should only be possible at λ < 235 nm;^10^ the apparent one-photon signal dependence observed in earlier laser-induced fluorescence studies likely resulted from facile saturation of the optical transition to the S_2_ state.

While Antonov et al.^10^ have comprehensively characterized the products resulting from AcAc photochemistry, the detailed mechanisms are still somewhat uncertain in the absence of measurements performed under collision-free conditions that can also quantify the photofragment translational energy release. As is well known for simple carbonyl species, Norrish type I pathways can occur statistically on S_0_ or over exit barriers on T_1_, leading to characteristic translational and internal energy distributions,^33−37^ while molecular dissociation via S_0_ transitions states or roaming pathways can also be distinguished.^38−40^ In this paper, we present the results of a velocity-map ion imaging study of AcAc photochemistry following UV excitation to S_2_ at 280 nm using single-photon VUV ionization to detect products. The experiments find evidence of primary photofragments that arise from Norrish type I dissociation on both triplet and singlet surfaces as well as products of dissociative ionization of primary photofragments.

## Experimental Methods

Experiments were performed using a DC slice velocity-map imaging apparatus that has been detailed previously,^36,41^ and only a brief description is provided here. The apparatus consists of source, ionization, and detector regions evacuated by turbomolecular pumps (Leybold 1100C, Pfeiffer 750M, and Leybold 360) backed by roughing pumps (Edwards XDS35ic, Leybold D65B) to base pressures of ∼1 × 10^–7^ Torr. During operation, the source region pressure increased to ∼10^–5^ Torr. Ar carrier gas at a pressure of ∼1 atm was bubbled through a liquid sample of AcAc (Acros Organics) in a stainless-steel finger, cooled in a water ice bath, and supersonically expanded into high vacuum by a solenoid valve (General Valve, Series 9) operating with 500 μs pulse duration. The expansion was skimmed to produce a molecular beam, which was intersected by counter-propagating and spatially overlapped UV and VUV laser beams. Based on the vapor pressure of AcAc at 273 K,^42,43^ the expansion is estimated to contain 0.2% AcAc.

UV pulses at 280 nm (4.43 eV) were produced by frequency-doubling in a BBO crystal the visible output of a Nd/YAG-pumped dye laser (Continuum ND6000 + Surelite III-10, 10 Hz repetition rate) operating with Rhodamine 590 laser dye. A double Fresnel rhomb (EKSMA Optics) was used to flip the horizontally polarized UV beam vertically, ensuring the ejected photofragments would have cylindrical symmetry about an axis parallel to the plane of the detector.^44^ The UV beam was loosely focused into the center of the ionization region of the apparatus by an anti-reflection coated fused silica lens with a focal length of *f* = 500 mm. The lens was positioned to ensure that the UV beam was unfocused at the molecular beam. The resulting UV beam diameter in the interaction region was ∼1 mm. The UV pulse energy could be varied from ∼10 μJ to ∼1 mJ (giving fluences ranging from ∼1.3 to ∼130 mJ cm^–2^) using combinations of neutral-density filters. VUV pulses at 118.2 nm (10.49 eV) were generated by frequency-tripling the 355 nm output of a Nd/YAG laser (Continuum Surelite II-10, 10 Hz repetition rate) in a Xe/Ar gas mixture. The 355 nm beam was attenuated using a λ/4 waveplate and Glan-Taylor polarizer to give pulse energies ∼8 mJ and focused near the center of the 57 cm long mixing cell by a UV-grade fused silica lens (*f* = 500 mm). A stable conversion was achieved with *P*_Xe_ = 25 Torr and *P*_Ar_ = 308 Torr, giving an Ar/Xe ratio of 12.3 that is consistent with previous studies.^45−47^ An internal MgF_2_ lens (*f* = 75 mm) acted as a window between the gas cell and the ionization region of the VMI apparatus and focused the VUV while leaving the residual 355 nm unfocused. We calculate beam diameters in the ionization region of ∼3 mm for the residual 355 nm and ∼80 μm for the 118 nm beam, giving fluences of 115 and 0.2 mJ cm^–2^, respectively. The latter value is estimated based on previously reported conversion efficiencies for 118 nm generation in Xe/Ar mixtures obtained using ns pulsed lasers.^47^ VUV generation required continuous convective mixing within the cell, which was maintained by cooling one limb of a circulating loop with an acetone/dry ice slush at 195 K.

Ions were accelerated toward a 40 mm diameter dual microchannel plate (MCP) and P46 phosphor screen detector assembly (Photonis). A fast high-voltage switch (Photek GM-MCP-2, gate duration ∼20 ns) was used to gate the detector to allow mass selection and DC slicing.^48^ Total phosphorescence was detected using a silicon photomultiplier (SenSL) coupled to a high-definition oscilloscope (LeCroy HDO4054), used to record time-of-flight mass spectra; the ∼60 ns lifetime of the phosphor limits the obtainable resolution, although this can be overcome to some extent by step scanning the detector gate over masses of interest. Ion images were captured by a complementary metal–oxide semiconductor (CMOS) camera (Basler acA1300-60gm). Synchronization of the pulsed valve, lasers, detector gate, and CMOS camera exposure was achieved using a digital delay generator (Quantum Composers, 9528). All data acquisition and experimental control were performed using software written in LabVIEW 2013 (National Instruments) and subsequently analyzed using procedures written in Igor Pro (WaveMetrics).

All ab initio calculations were performed using the GAMESS package (version 2020 R2).^49^ Geometry optimizations and harmonic frequency calculations were performed for various stationary points on the S_0_ surface using the B3LYP functional with Dunning’s cc-pVDZ basis set. Exclusively real harmonic frequencies confirmed that the optimized geometries of either tautomer of AcAc and molecular or radical products were local minima on the potential energy surface. All transition states identified had only one imaginary frequency, confirming that they were first-order saddle points.

## Results

Excitation at 280 nm prepares the S_2_ state of enol-AcAc almost exclusively. The enolone is present at an almost 20 times higher concentration than the diketone, and the transition to the S_2_ state has an oscillator strength that is more than 30 times greater than the transition to the S_1_ state of the minor diketone form.^8^ Direct excitation to the S_1_(nπ*) state of enol-AcAc may also occur in this wavelength region^21^ with a cross section that is more akin to typical α,β-unsaturated carbonyls such as MVK, although this possibility appears not to have been considered in the previous spectroscopic analyses. Regardless, IC from S_2_ → S_1_ is expected to occur on a sub-ps timescale,^18−20^ which is effectively instantaneous on the nanosecond timescale of the current experiments.

### Time-of-Flight Mass Spectra

The ionization energy of enol-AcAc is 8.85 and ∼9.5 eV for the diketone form.^10,50,51^ The 118.2 nm (10.49 eV) VUV beam can cause dissociative ionization of both tautomers.^10^ One-color VUV-only mass spectra show primarily the AcAc^+^ parent ion at *m*/*z* = 100 with a weaker feature at *m*/*z* = 85 that results from dissociative ionization of the dominant enolone tautomer. Dissociative ionization of the minor diketone tautomer produces ions with *m*/*z* = 43, 58, and 72,^10^ but VUV-only signal at these masses is difficult to discern within the experimental signal-to-noise ratio, partially due to interference from ionization of background gas within the chamber. The 280 nm UV beam does not produce ions to any significant extent at pulse energies <0.5 mJ (<64 mJ cm^–2^). VUV generation requires convection in the tripling cell—without the acetone/dry ice slush, the unfocused residual 355 nm causes no ionization, either with or without the presence of the UV beam.

**Figure 2:**
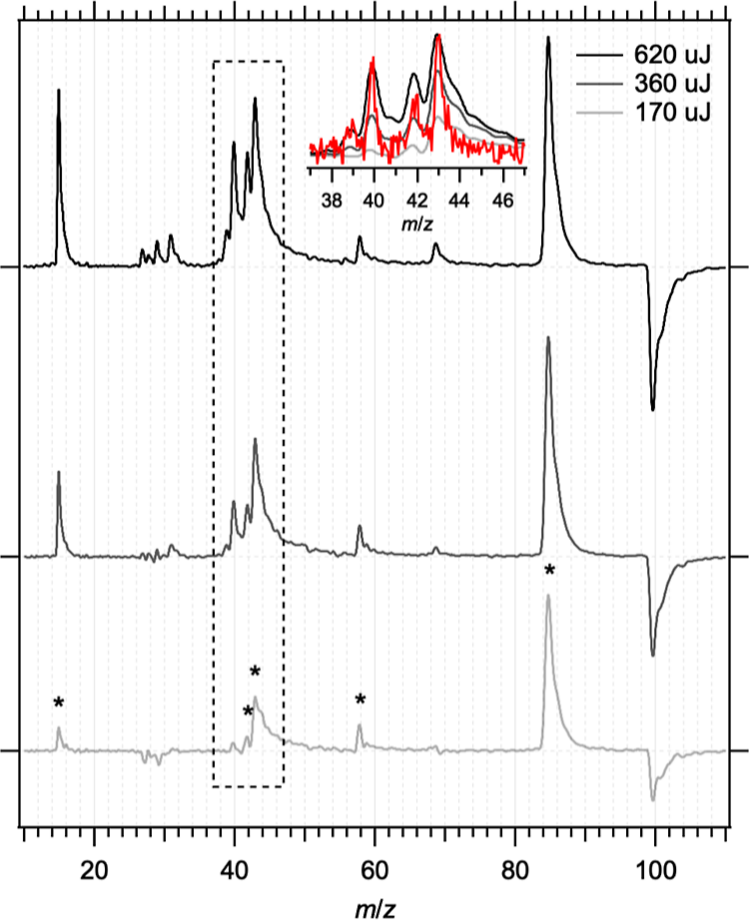
Time-of-flight mass spectra for AcAc after background subtraction [280 + 118]—[118] at various 280 nm pulse energies at 20 ns photolysis-probe delay. Ion images were collected for features marked with an asterisk. The inset shows a higher-resolution mass spectrum of the *m*/*z* = 37–47 region obtained by scanning the detector gate.

The time-of-flight mass spectra in Figure 2 show the two-color UV + VUV contribution after subtraction of the VUV-only mass spectra, i.e., [UV + VUV] – [VUV], acquired at three different UV laser pulse energies (170, 320, and 620 μJ corresponding to fluences of 22, 41, and 79 mJ cm^–2^, respectively). The time delay between the UV and VUV beams was 20 ns; measurements of the time profiles as the UV-VUV delay varied are summarized in Figure S2 of the Supporting Information. The AcAc^+^ ion at *m*/*z* = 100 is depleted when the UV beam is introduced at earlier times, indicating loss of the neutral species. The magnitude of the depletion is ∼20–60% of the VUV-only peak, across the range of UV photolysis pulse energies shown. The presence of the UV beam also leads to additional fragment peaks at *m*/*z* ≤ 85. The major two-color fragments at lower UV energies give peaks at *m*/*z* = 15, 42, 43, 58, and 85 in the mass spectrum, which are indicated with asterisks in Figure 2. The mass spectra become increasingly congested as the UV pulse energy is increased and additional masses appear. A consequence of the strength of the S_0_ → S_2_ transition is that it is readily saturated at modest UV pulse energies, which increases the likelihood of multiphoton dissociation.^10^ Measurements of the dependences of the *m*/*z* = 15, 42, 43, and 85 signals on the UV pulse energy over the range 40–420 μJ (fluence 5–54 mJ cm^–2^) are summarized in Figure S4 and Table S1 of the Supporting Information. The results are somewhat inconclusive but suggest that there may be a contribution from multiphoton excitation at higher pulse energies to the *m*/*z* = 15 and 42 fragments (*n* > 1). In contrast, the *m*/*z* = 43 and 85 signals increase more slowly with UV fluence (*n* < 1).

The detector phosphorescence lifetime limits the mass resolution. For sequential masses, those arriving later may be lost in the phosphorescence decay of an earlier arriving mass especially if less abundant. Using a very short detector gate and stepping over the arrival times allows the individual mass peaks to be identified more clearly. The inset in Figure 2 shows an example of a detector gate scan (after subtraction of one-color VUV-only background signal), where *m*/*z* = 39, 40, 42, and 43 are clearly resolved. While the *m*/*z* = 39 and 40 mass peaks are very weak at low UV laser fluences, their intensity increases dramatically as the fluence is increased, suggesting that they result from multiphoton dissociation. Similar gate scans over other regions show that the features at *m*/z = 15, 58, and 85 are isolated with no significant two-color signal in nearby mass channels.

We focus on the major features evident in the two-color UV + VUV mass spectra observed at the lowest UV pulse energies, namely *m*/*z* = 15, 42, 43, 58, and 85, for which ion images (discussed below) were recorded. While these ions may originate from UV photolysis of AcAc with subsequent VUV ionization of the neutral fragment, there are other potential sources. Dissociative ionization of energized ground-state AcAc S_0_* following VUV absorption may directly produce ionic photofragments. The total excitation energy in that case is 14.92 eV (280 + 118 nm), which is comfortably greater than the appearance energies of all the fragment ions detected, although the persistence of signals at delay times that extend beyond the expected AcAc S_0_* lifetime of <50 ns (discussed below, see also Figure S2) suggests that this mechanism is of minor importance, even at a relatively short delay of 20 ns. Neutral primary photoproducts may also undergo dissociative ionization, leading to the formation of lower-mass daughter ions.

The *m*/*z* = 15 fragment can be immediately assigned to CH_3_^+^, which can be produced by VUV photoionization of neutral methyl radicals (CH_3_, IE = 9.84 eV)^52^ resulting from Norrish type I dissociation of either the enolone or diketone tautomer
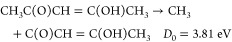
1a
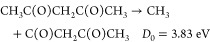
1b

The CBS-QB3 calculations performed by Antonov et al.^10^ have found very similar dissociation energies *D*_0_ for pathways (1a) and (1b), confirming that both are energetically accessible at 280 nm (*E*_hν_ = 4.43 eV). Ionization energies for both larger radical products are expected to be <8 eV, based on ab initio calculations at the B3LYP/cc-pVDZ level, and thus, they can be photoionized by the VUV pulse, though the different *m*/*z* = 85 radical cations cannot be distinguished by VUV ionization. The magnitude of the *m*/*z* = 85 signal indicates a photoionization cross section that is around 6–8 times larger than that of CH_3_ at 118 nm (∼6 Mb).^53,54^ At higher UV pulse energies, the *m*/*z* = 15 signal increases more rapidly than the *m*/*z* = 85 signal (see power dependence measurements in Figure S4 and Table S1 of the Supporting Information). The *n* > 1 photon dependence for *m*/*z* = 15 indicates that there may be a contribution that arises from multiphoton excitation. In contrast, the *m*/*z* = 85 channel has a photon dependence *n* < 1, suggesting that secondary absorption of the UV beam and dissociation may limit the overall yield. The ion imaging experiments described below strongly suggest that the *m*/*z* = 15 and 85 fragments are primarily formed photolytically as a neutral radical pair. Both *m*/*z* = 15 and *m*/*z* = 85 photofragments show appearance times (Figure S2) that are within the instrument resolution, limited by the 6 ns pulse duration of the laser systems.

The alternative Norrish type I dissociation pathway leading to the formation of acetyl radicals (CH_3_CO, IE = 7.0 eV)^52^ can also occur from either the enolone or diketone tautomer
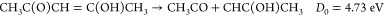
2a
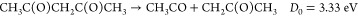
2b

At 280 nm, however, only reaction 2b forming the acetonyl radical CH_2_C(O)CH_3_ as the co-product is energetically accessible based on the dissociation energies calculated at the CBS-QB3 level.^10^ Subsequent VUV ionization would give rise to peaks at *m*/*z* = 43 (CH_3_CO^+^) and *m*/*z* = 57 (CH_2_C(O)CH_3_^+^), although only the former is evident in the mass spectra shown in Figure 2. The absence of any *m*/*z* = 57 signal may be attributed to dissociative ionization of acetonyl leading to *m*/*z* = 29, although that mass channel is evident mainly at higher UV pulse energies (see Figure 2). CH_3_CO has a photoionization cross section of ∼11 Mb at 118 nm;^10,41^ therefore, the magnitude of the *m*/*z* = 43 signal compared to *m*/*z* = 15 may suggest broadly similar branching between the two Norrish type I pathways. However, dissociative ionization, likely of the C_4_H_5_O_2_ primary product, cannot be conclusively ruled out as a source for the *m*/*z* = 43 ions observed. The dependence of the *m*/*z* = 43 signal on delay time can be seen in Figure S2. The short-time behavior is complicated near zero time by reversal of the UV and VUV laser pulses—UV photolysis of AcAc^+^ cations leads primarily to *m*/*z* = 43 photofragments—but seems to indicate that the appearance time is primarily instrument limited. An additional slow component appears to grow in on a timescale of ∼100 ns.

The *m*/*z* = 42 and 58 peaks in the mass spectra can result from photoionization of the ketene and acetone/propen-2-ol molecular products of unimolecular dissociation on the S_0_ surface
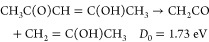
3a
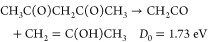
3a′
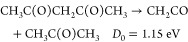
3b

Two energetically accessible pathways exist for reactions 3a and 3a′, which can occur via TSs on S_0_ that connect the products to both enol-AcAc and keto-AcAc. A single TS connecting the keto-AcAc has been identified for reaction 3b. The molecular products ketene (CH_2_CO, IE = 9.62 eV), acetone (CH_3_C(O)CH_3_, IE = 9.70 eV), and propen-2-ol (CH_2_=C(OH)CH_3_, IE = 8.58 eV) can all be photoionized at 118 nm.^52^ The photoionization cross section of ketene is relatively large at 118 nm (24.8 Mb),^55^ and the weak signal observed at *m*/*z* = 42 at low UV pulse energies would suggest a small yield for this product if it were formed via unimolecular dissociation on the S_0_ surface. However, the time profiles, ion imaging results, and RRKM rate calculations described below suggest that the *m*/*z* = 42 and 58 fragments are most likely not formed by unimolecular dissociation on S_0_ but are more likely to result from dissociative ionization of the C_4_H_5_O_2_ primary photoproduct.

### Velocity-Map Ion Images

**Figure 3:**
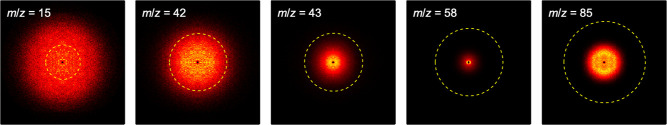
Symmetrized ion images for *m*/*z* = 15, 42, 43, 58, and 85. Each image is the average of three independent measurements after subtraction of one-color VUV-only background signals. The dashed circle in each panel corresponds to a speed of 1000 m s^–1^.

**Figure 4:**
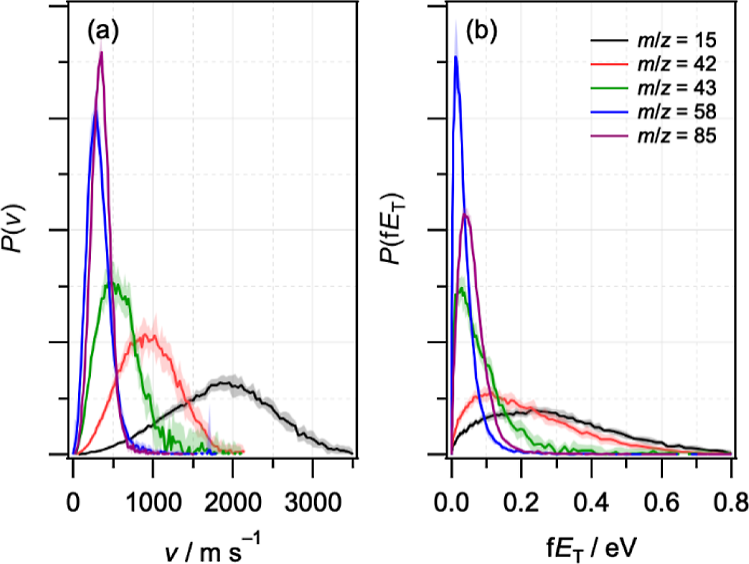
Normalized (a) speed distributions *P*(*v*) and (b) fragment translational energy distributions *P*(f*E*_T_) for *m*/*z* = 15, 42, 43, 58, and 85 fragments.

Figure 3 shows symmetrized DC sliced ion images of the two-color UV + VUV fragments at *m*/*z* = 15, 42, 43, 58, and 85, following subtraction of one-color VUV-only background signals. All two-color UV + VUV and one-color VUV ion images were recorded in triplicate (30,000 ions per image). Ion images recorded at low UV laser pulse energies (∼60 μJ, corresponding to a fluence of ∼8 mJ cm^–2^) to minimize contributions from multiphoton processes produced identical speed distributions (see Figure S3 in the Supporting Information). Fragment radial and angular distributions for each mass were extracted directly from the individual images and averaged. The images appear isotropic, and fitting the angular distributions to the expression *P*(θ) ∝ β*P*_2_(cos θ) yields values of the anisotropy parameter β that are close to zero.^44,56^ Radial distributions were converted into speed distributions using a calibration factor determined from ion images of the I/I* products of CH_3_I photolysis (see the Supporting Information for details), with an estimated uncertainty of ∼2%. The speed and fragment translational energy distributions, *P*(*v*) and *P*(f*E*_T_), are shown in Figure 4, and the average values are summarized in Table 1.

**Table 1:** Summary of Results from Ion Image Analysis: Average Speeds, Average Fragment Translational Energies, and Speed-Averaged Anisotropy Parameters

*m*/*z*	Fragment	⟨*v*⟩/m s^–1^	⟨f*E*_T_⟩/eV	⟨β⟩
15	CH_3_	1860	0.30	+0.03
42	CH_2_CO (ketene)	950	0.23	+0.03
43	CH_3_CO (acetyl)	570	0.09	+0.00
58	C_3_H_6_O (acetone or propen-2-ol)	330	0.04	+0.03
85	C_4_H_5_O_2_	350	0.06	+0.02

**Figure 5:**
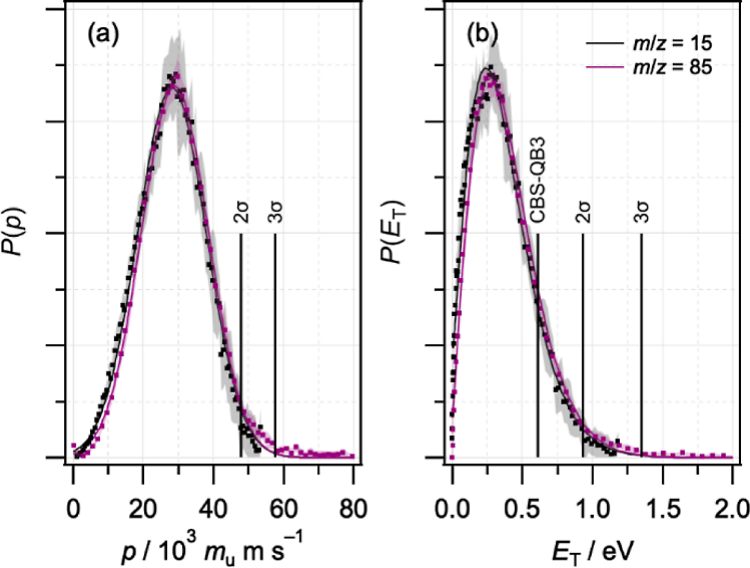
Normalized (a) momentum distributions *P*(*p*) and (b) total translational energy distributions *P*(*E*_T_) for *m*/*z* = 15 and 85 fragments (dots). Solid lines are Gaussian fits to the experimental *P*(*p*) distributions in (a) and the resulting *P*(*E*_T_) distributions in (b). Vertical lines indicate *p*_max_ values determined at ⟨*p*⟩ + 2σ (solid) and ⟨*p*⟩ + 3σ (dashed), as indicated, and resulting *E*_T,max_ values. *E*_T,max_ determined from dissociation energy calculated at the CBS-QB3 level^10^ is also shown.

The *m*/*z* = 15 and 85 ions can, in principle, be produced by VUV ionization of the neutral products of a Norrish Type I mechanism. Figure 5 shows that the approximately Gaussian momentum distributions *P*(*p*) for these ions are identical, confirming that momentum-matched neutral methyl CH_3_ and the associated C_4_H_5_O_2_ radicals are formed originally via either reaction 1a or 1b. For this product pair, the dissociative ionization mechanism can be definitively ruled out. Total kinetic energy distributions *P*(*E*_T_), also shown in Figure 5, are obtained from the *P*(*v*) distributions for *m*/*z* = 15 and 85 using
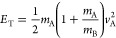
E1where the labels A and B indicate the detected and undetected fragments, respectively. The *P*(*E*_T_) distributions derived in this way are identical as expected, with average ⟨*E*_T_⟩ = 0.38 eV and extending to a maximum translational energy *E*_T,max_ ≈ 1 eV. As noted earlier, the CBS-QB3 calculations reported by Antonov et al.^10^ suggest nearly identical dissociation energies for reactions (1a) and (1b) with an average *D*_0_ = 3.82 eV. The 280 nm UV photon provides *E*_hν_ = 4.43 eV, limiting the translational energy to *E*_T_ ≤ 0.61 eV, according to the energy conservation equation
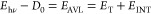
E2

Approximately one-third of the CH_3_ + C_4_H_5_O_2_ radical product pairs are formed with *E*_T_ ≥ 0.61 eV, suggesting that the true *D*_0_ value is smaller than that predicted by theory. We identify the maxima in the momentum distributions for each fragment by fitting it to a Gaussian function and determining the cutoff *p*_max_ to be at least 2σ beyond the mean, which captures around 95% of the distribution. Using this value as a cutoff provides an upper limit of *D*_0_ ≤ 3.49 eV, with an estimated uncertainty of 0.13 eV. The fraction of the available energy partitioned into translation is *f*_T_ ≥ 41%. Previously, we found similar *f*_T_ values for Norrish type I dissociation of acetaldehyde and acetone on the T_1_ surface when the available energy was ∼0.5 eV above the T_1_ barrier.^36,41^ In contrast, barrierless dissociation on S_0_ is statistical and results in slow-moving fragments with far smaller values of *f*_T_.^33−37^

The alternative energetically accessible Norrish type I pathway, reaction 2b via the diketone tautomer, leads to acetyl CH_3_CO and acetonyl CH_2_C(O)CH_3_ fragments that would appear at *m*/*z* = 43 and *m*/*z* = 29 (the parent ion, which would appear at *m*/*z* = 57, is not observed as a result of dissociative ionization^10^) in the mass spectrum, respectively. Only the former is clearly observed as noted above. Assuming the formation of a neutral acetyl + acetonyl product pair, the total translational energy distribution *P*(*E*_T_) has ⟨*E*_T_⟩ = 0.16 eV. Based on the reported CBS-QB3 dissociation energy, *E*_AVL_ = 1.10 eV; the fraction of available energy partitioned into translation *f*_T_ = 14% is far smaller than the *f*_T_ observed for methyl radicals formed via either reaction 1a or 1b and would be consistent with barrierless dissociation on S_0_. Dissociative ionization of the thermalized diketone tautomer has been shown to lead to the formation of *m*/*z* = 43 along with *m*/*z* = 58 and 72 fragments.^10^ In the absence of collisions to stabilize any diketone produced via phototautomerization, the absence of *m*/*z* = 72 fragments in the mass spectra is expected.

Finally, the obvious assignment for the *m*/*z* = 42 fragment is ketene, which can be formed along with either acetone or its less stable isomer propen-2-ol, as shown in reactions (3a) and (3b). Both reactions were identified as minor one-photon dissociation channels (Φ < 4%) by Antonov et al.^10^ The reverse barrier heights for each of these unimolecular dissociation pathways on S_0_ range from ∼1 to 3 eV, suggesting that they may be distinguishable in the ion images even in the absence of species- or state-resolved detection. However, the speed distributions show they are not formed as neutral co-fragments. For the *m*/*z* = 15 and 85 pair, the ratio of average speeds ⟨*v*_15_⟩/⟨*v*_85_⟩ = 5.6 (see Table 1) is approximately the same as the inverse mass ratio *m*_85_/*m*_15_ = 5.7, and the *P*(*p*) distributions in Figure 5 are the same, confirming that they are formed as momentum-matched neutrals. In contrast, the ratio ⟨*v*_42_⟩/⟨*v*_58_⟩ = 2.9 is around twice the inverse mass ratio and the momentum distributions are very different (see Figure S5 in the Supporting Information).

## Discussion

There are some differences between the two-color difference mass spectra for 280 nm excitation shown in Figure 2 and those reported by Antonov et al.^10^ at shorter photolysis wavelengths of 248 and 266 nm. Following UV excitation at 266 nm and using a similar 10.45 eV VUV photon for ionization, fragments at *m*/*z* = 15, 29, 40, 42, 43, 58, 72, 82, and 84 were identified (see Figure S3 in the Supporting Information of Antonov et al.), while we observe *m*/*z* = 15, 40, 42, 43, 58, and 85 when using low UV pulse energies to minimize multiphoton dissociation. The *m*/*z* = 40 signal shows a very strong dependence on UV pulse energy and is likely a product of multiphoton or secondary dissociation. Ions with *m*/*z* = 82 and 84, previously assigned to acetyl allene and acetyl ketene, formed in conjunction with water and methane, respectively, are not observed at 280 nm although they are in principle detectable using 10.49 eV VUV radiation. The mass spectra in Figure 2 display only a peak at *m*/*z* = 85 in this region, which previously was observed only as the product of dissociative ionization of the enolone tautomer. The strong two-color [UV + VUV] enhancement of the signal and the momentum-matched *m*/*z* = 15 co-fragment indicates that it is formed as a neutral radical.

The origin of the apparent differences from the results of Antonov et al.^10^ is not entirely clear, but we note that there are several differences in the experimental conditions that may be collectively responsible. The most obvious difference is that the ion imaging experiments are performed under collision-free conditions rather than in the collisional environment of the flow cell. Additionally, the excitation wavelengths (280 nm versus 248 nm or 266 nm) and the internal state distributions of the AcAc prior to excitation (thermal versus jet-cooled) may access different regions of the excited-state potential energy surfaces, and result in different dynamics.

**Figure 6:**
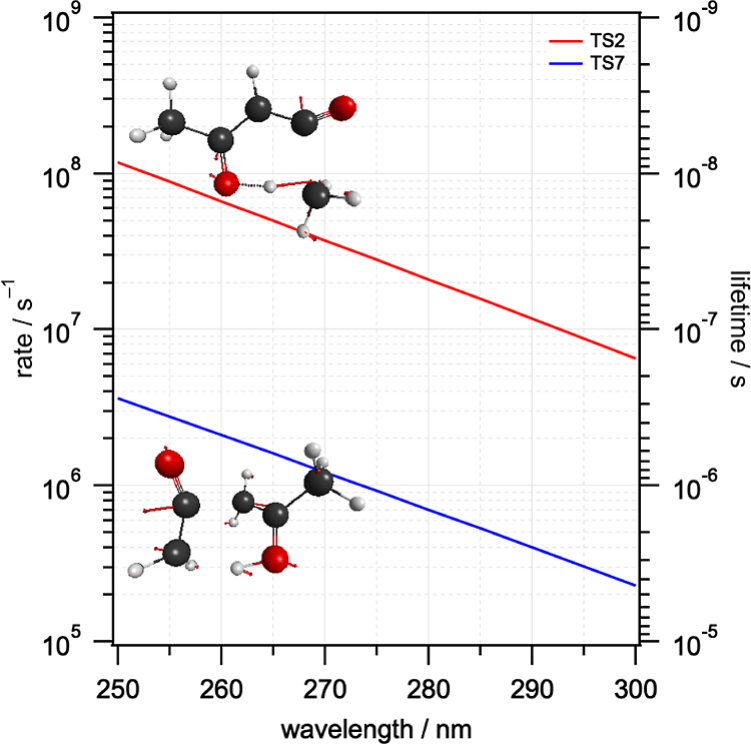
RRKM rates and lifetimes as a function of excitation wavelength for the dissociation of enol-AcAc via TS2 to form methane + acetyl ketene and keto-AcAc via TS7 to form ketene + propen-2-ol. TS geometries calculated at the B3LYP/cc-pVDZ level are also shown, with red arrows indicating the displacements associated with the imaginary mode.

Following excitation of AcAc to the S_2_ state, the population is returned to S_0_ via a series of non-radiative steps within ∼330 ps.^17−20^ In this work, the VUV ionization pulse was introduced 20 ns after UV excitation—all product ions were observed to form on a timescale faster than the laser pulse durations (<6 ns, see Figure S2). An upper-limit lifetime of energized AcAc S_0_* can be estimated using RRKM calculations.^57^ Using the Antonov et al.^10^ CBS-QB3 calculations as a template, we have performed ab initio calculations to optimize the geometries and calculate the harmonic vibrational frequencies of the seven dissociation transition states (labeled TS2–TS8, see Table 2) that lead to molecular products. The optimized geometries at the B3LYP/cc-pVDZ level of theory are very similar to those reported previously [the CBS-QB3 composite method uses B3LYP/6-311G(2d,d,p) geometries], and the overall energies agree reasonably well with the CBS-QB3 values, which are summarized in Table 2. The TS coordinates and harmonic frequencies are compiled in the Supporting Information. Vibrational densities and sums of states were calculated using the Whitten–Rabinovitch approximation^58^ and used to estimate the unimolecular dissociation rates at a total energy *E* using the standard RRKM expression
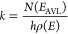
E3*N*(*E*_AVL_) is the sum of states evaluated at *E*_AVL_ = *E*–*E*_TS_ for the relevant transition state, and ρ(*E*) is the density of states evaluated at *E* for either enol-AcAc or keto-AcAc. The calculated RRKM rates and associated lifetimes with total energy *E* = 35,710 cm^–1^ (280 nm) are compiled in Table 2. The RRKM rates vary by 10 orders of magnitude. Dissociation of S_0_ via TS2, which leads from enol-AcAc to methane + acetyl ketene, dominates the total loss rate and would account for 97% of products formed via the molecular TSs on S_0_. The second-fastest process, dissociation of keto-AcAc via TS7 to form ketene + propen-2-ol, is more than an order of magnitude slower and would make up the remaining 3% of S_0_ unimolecular dissociation products. The other molecular pathways contribute negligibly. The wavelength dependence of the RRKM dissociation rates and lifetimes for TS2 and TS7 are shown in Figure 6. Overall, the dissociation rate after excitation at 280 nm via the molecular transition states TS2–TS8 is estimated to be 2.2 × 10^6^ s^–1^, which would result in a relatively long S_0_ lifetime of <46 ns. This lifetime does not include contributions from barrierless radical–radical processes (e.g., those that lead to the Norrish type I products as shown in R1a, R1b, and R2b) for which variational calculations would be required. Consequently, the calculated S_0_ lifetime of 46 ns should be regarded as an upper limit that will be reduced by contributions from barrierless pathways, which are likely to be competitive.^59^

**Table 2:** Ab Initio Energies for TSs Forming Molecular Products on S_0_ [All Values Are Δ(*E* + ZPE) at 0 K]^a^

	products	Δ(*E* + ZPE)/eV	*k*/s^–1^	τ/s
enol-AcAc		0.00 (0.00)		
keto-AcAc		0.27 (0.13)		
TS1	keto-enol tautomerization	2.49 (2.58)		
TS2	methane + acetyl ketene	2.34 (2.58)	2.1 × 10^7^	4.8 × 10^–8^
TS3	methane + hydroxyvinyl ketene	3.73 (3.83)	0.16	6.1
TS4	water + acetyl propyne	3.88 (3.82)	0.10	9.9
TS5	water + acetyl allene	3.99 (4.05)	4.9 × 10^–4^	2.3 × 10^2^
TS6	ketene + propen-2-ol	3.59 (3.65)	1.5	0.67
TS7	ketene + propen-2-ol	2.42 (2.49)	7.0 × 10^5^	1.4 × 10^–6^
TS8	ketene + acetone	3.80 (3.90)	0.30	3.3

a Results of B3LYP/cc-pVDZ (this work) and CBS-QB3 (Antonov et al.,^10^ in parentheses) calculations are shown. Transition states TS2–TS6 connect enol-AcAc to products, while TS7 and TS8 connect keto-AcAc to products. Calculated RRKM rates *k* and lifetimes τ are also shown.

According to the RRKM calculations, the methane + acetyl ketene pathway, which occurs via TS2, should dominate the unimolecular dissociation of S_0_*, but was found by Antonov et al. to have a photofragment quantum yield of only Φ = 0.12. The VMI experiments described here are insensitive to methane (IE = 12.61 eV),^52^ and we see no evidence of acetyl ketene (IE = 9.59 eV)^10^ at *m*/*z* = 84. The most significant photolytic product channel identified by Antonov et al. leads to water + acetyl allene (IE = 12.62 and 9.48 eV, respectively)^10,52^ with a quantum yield of Φ = 0.25. This reaction occurs on S_0_ via TS4 but has an exceptionally slow RRKM rate constant of only 0.10 s^–1^ and a lifetime of 9.9 s and consequently should have a negligible yield. While the current experiments are insensitive to water, the absence of any signal at *m*/*z* = 82 is unsurprising if the unimolecular dissociation rate were to be as slow as the RRKM calculations predict. At shorter excitation wavelengths, the unimolecular dissociation rates increase, as shown in Figure 6, but passage over TS2 continues to dominate.

Although the *m*/*z* = 42 and 58 fragments appear to form energetically accessible product pairs (ketene + acetone or ketene + propen-2-ol), they are formed with distinctly different momentum distributions, indicating that they are not co-fragments. The RRKM rates for formation of ketene + acetone via TS8 and ketene + propen-2-ol are both exceptionally slow (see Table 2) implying that branching into these channels should be insignificant. Dissociation via TS7 leads from keto-AcAc to ketene + propen-2-ol with a significantly longer lifetime of 1.4 μs; however, this results in a branching fraction of only 3%. One possible source of the *m*/*z* = 42 and 58 fragments is dissociative ionization of energized AcAc S_0_*. Using known ionization energies for ketene, acetone, and propen-2-ol,^52^ and *D*_0_ values for neutral dissociation from the CBS-QB3 calculations,^10^ the threshold appearance energies are in the range 10.4–11.3 eV, substantially lower than the total energy after absorption of both UV and VUV photons of 14.92 eV. However, the time profiles for the *m*/*z* = 42 and *m*/*z* = 58 fragments (Figure S2) are inconsistent with the expected lifetime of AcAc S_0_* of <46 ns, except at very short delays. Secondary UV photolysis of C_4_H_5_O_2_ radical primary photoproducts followed by VUV ionization or direct dissociative ionization may be the source of these fragments.

The *m*/*z* = 15 and 85 radical pair are formed as momentum-matched neutral CH_3_ and either C(O)CH=C(OH)CH_3_ or C(O)CH_2_C(O)CH_3_ radicals that are subsequently ionized by the VUV pulse, via a Norrish type I dissociation of either enol-AcAc or keto-AcAc shown in reactions (1a) and (1b). Ab initio calculations at both the CBS-QB3 and B3LYP/cc-pVDZ levels predict near-identical dissociation energies. The current experiments cannot conclusively distinguish between the two *m*/*z* = 85 species, but the relatively large fraction of the available energy partitioned into relative translation (*f*_T_ ≥ 0.41) is consistent with dissociation over a barrier on a triplet surface with a relatively large excess energy, as observed previously for the photolysis of the monocarbonyls acetaldehyde and acetone.^36,41^ Consequently, we conclude that following initial excitation, the system evolves non-radiatively as deduced by the ultrafast studies S_2_ → S_1_ → T_1_, whereupon a subset of the population can undergo dissociation over a barrier to form CH_3_ + C(O)CH=C(OH)CH_3_, while the remainder undergoes ISC to repopulate S_0_. The dissociation rate on T_1_ would have to be competitive with that for T_1_ → S_0_ ISC.^17,20^ The absence of the major S_0_ products predicted by the RRKM calculations (methane + acetyl ketene via TS2) suggests that dissociation on T_1_ may dominate under collision-free conditions. Relaxed reaction path calculations leading to CH_3_ elimination by Squibb et al.^19^ suggest that a barrier of 4.54 eV exists on the T_1_(ππ*) surface, which is only slightly greater than the available energy. Further ab initio calculations to locate and better characterize the TS for Norrish type I dissociation on the T_1_ surface would be valuable. Experimentally, measurements at longer UV wavelengths would also test the proposed mechanism. As the excess energy above the triplet barrier decreases, partitioning to internal motion should also decrease, leading to an increase in *f*_T_,^60^ as has been observed previously for acetaldehyde and acetone.^36,41^

The assignment of the *m*/*z* = 43 fragment to CH_3_CO formed via the alternative Norrish type I reaction 2b is tentative as the expected acetonyl co-fragment undergoes dissociative ionization to give *m*/*z* = 29 products, which appeared only as an exceptionally weak signal in the mass spectra. Assuming acetyl + acetonyl is formed, a far smaller fraction of the available energy is partitioned into relative translation (*f*_T_ = 0.15), which suggests that dissociation occurs without a barrier. The most likely mechanism following excitation to S_2_ is non-radiative population transfer back to S_0_ on a sub-nanoscale timescale, followed by phototautomerization to keto-AcAc and subsequent barrierless dissociation, as initially proposed by Antonov et al.^10^ The enolone tautomer cannot dissociate to form acetyl radicals at 280 nm. Based on known photoionization cross sections for CH_3_ and CH_3_CO, the two Norrish type I dissociation pathways leading to their formation appear to have qualitatively similar branching fractions. Alternative mechanisms cannot be conclusively ruled out, however. As the time profile would appear to eliminate dissociative ionization of AcAc S_0_*, except at very short delays, and the *n* < 1 photon dependence argues against secondary UV dissociation, the most probably alternative to Norrish type I dissociation on S_0_ appears to be dissociative ionization of the C_4_H_5_O_2_ primary photoproduct.

Rowell et al.^61,62^ have discussed the relative branching between Norrish type I pathways and other dissociation mechanisms for a broad range of organic carbonyls relevant to atmospheric chemistry. While Norrish type I dissociation on S_0_ is available to all carbonyls, the accessibility of the T_1_ mechanism is limited by functionality. Cleavage of either α-bond adjacent to the C=O moiety can occur, forming either larger or smaller alkyl radical products, labeled type Ia and Ib, respectively. In α,β-unsaturated carbonyls, the intrinsic barrier for type Ia dissociation is increased by resonance stabilization, rendering that pathway inaccessible at actinic wavelengths, as demonstrated for both acrolein and MVK. However, the increase in barrier height is not seen for type Ib dissociation, which remains accessible under tropospheric conditions. This trend is consistent with our assignment of dissociation on T_1_ for the *m*/*z* = 15 and 85 radical pair in AcAc (type Ib). In contrast, tautomerization is a competitive ground-state pathway for α,β-unsaturated carbonyls. Following tautomerization, it is plausible that keto-AcAc would dissociate via the type Ia mechanism to produce *m*/*z* = 43 and 57 fragments, as is tentatively assigned here. The proposed photolysis pathways of AcAc are accordingly consistent with the known tropospheric photochemistry of both mono- and α,β-unsaturated carbonyls.

## Conclusions

Velocity-map ion imaging coupled with VUV ionization has been used to study the UV photochemistry of enol-AcAc following excitation to S_2_ at 280 nm. The major fragment mass peaks observed were *m*/*z* = 15, 42, 43, 58, and 85. All ions were formed on a timescale that was shorter than the laser pulse duration (<6 ns) and produced isotropic images, indicating that the timescale for dissociation was longer than the rotational period of the parent molecule. The *m*/*z* = 15 and 85 fragments were momentum-matched and assigned to the Norrish type I neutral products CH_3_ + C(O)CH=C(OH)CH_3_ resulting from dissociation of enol-AcAc. The fraction of available energy partitioned into relative translation *f*_T_ = 0.41 suggests that dissociation occurs on the T_1_(ππ*) surface. The observed speed distributions were used to determine an upper-limit dissociation energy *D*_0_ ≤ 3.49 eV. The *m*/*z* = 43 fragment was tentatively assigned to the alternative Norrish type I product CH_3_CO, presumably formed in conjunction with CH_2_C(O)CH_3_, although this fragment was not directly detected. The smaller *f*_T_ value of 0.15 is consistent with dissociation of keto-AcAc following phototautomerization on S_0_. Alternatively, the time and power dependence of the *m*/*z* = 43 signal may indicate that it appears as a daughter ion following dissociative ionization, most probably, of the C_4_H_5_O_2_ primary photofragment. The *m*/*z* = 42 and 58 fragments were not momentum-matched, indicating that they were not formed as a product pair via unimolecular dissociation on S_0_. Similar dissociative ionization mechanisms are most likely responsible.

## Supporting Information Available

Spectrometer calibration data; photofragment time profiles; photofragment UV power dependences; photofragment momentum distributions; ab initio transition-state geometries; and harmonic vibrational frequencies (PDF)
