# Supplementary material for: Acetylacetone Photolysis at 280 nm Studied by Velocity-Map Ion Imaging
Source: J Phys Chem A. 2023 Aug 3;127(32):6687–96. doi: 10.1021/acs.jpca.3c01653 (PMC10440790; doi:10.1021/acs.jpca.3c01653)
Supplement: Supplementary file 1 — jp3c01653_si_001.pdf [file jp3c01653_si_001.pdf]

Supporting Information: Acetylacetone Photolysis at 280 nm Studied by Velocity-Map Ion Imaging

Johanna E. Rinaman and Craig Murray*

Department of Chemistry, University of California, Irvine, Irvine CA 92697, USA

* Email: craig.murray@uci.edu; Telephone: +1-949-824-4218

Spectrometer calibration

The velocity-map imaging spectrometer was calibrated using the well-known UV photolysis of CH_3I at 280 nm with VUV ionization detection of the I($^2P_{3/2}$) and I*($^2P_{1/2}$) atomic products.[1–4]

$$CH_3I \rightarrow CH_3 + I \qquad D_0 = 2.39 \text{ eV} \qquad \text{(S1a)}$$

$$CH_3I \rightarrow CH_3 + I^* \qquad D_0 = 3.33 \text{ eV} \qquad \text{(S1b)}$$

The uncertainty in the dissociation energies (1σ) is 0.03 eV (~1%).[5] Both I and I* products can be readily ionized at 118 nm. The photoionization cross section is approximately 20 times greater for I than I*, as a result of an accidental resonance with an autoionizing Rydberg state.[6] Figure S1 shows a typical symmetrized DC slice ion image. Ion counts near the center of the image are an experimental artefact. The ion image shows three distinct rings. The outermost and innermost rings can be assigned to I and I* atoms, respectively, formed in conjunction with CH_3 radicals in the vibrational ground sate. The intermediate feature corresponds to formation of I atoms with vibrationally excited $CH_3(\nu_1 = 1)$. The angular distributions are consistent with a parallel transition to the $^3Q_0$ state of the parent CH_3I molecule.

The radial distribution, also shown in Figure S1 is obtained by conversion to polar coordinates and direct integration over the polar angle, weighted by an $r \sin\theta$ Jacobian. Photofragment speeds v are related to the image radius r in pixels by the equation

$$v = k\frac{r}{t_{\mathrm{TOF}}}$$

where τ_{TOF} is the flight time for the m/z = 127 ion (11.34 μs) and k is a calibration factor. In order to account for internal (rotational) excitation of the undetected CH_3I fragment, we determine the cutoff in the radial distributions as r_0+2σ, where r_0 is the center of a Gaussian fit and σ the standard deviation. A pixel-to-speed calibration factor k = (36.6±0.6) m s^{-1} μs pixel^{-1} results in excellent

agreement with the expected maximum speeds based on the known dissociation energies for all three features present in the radial distribution.

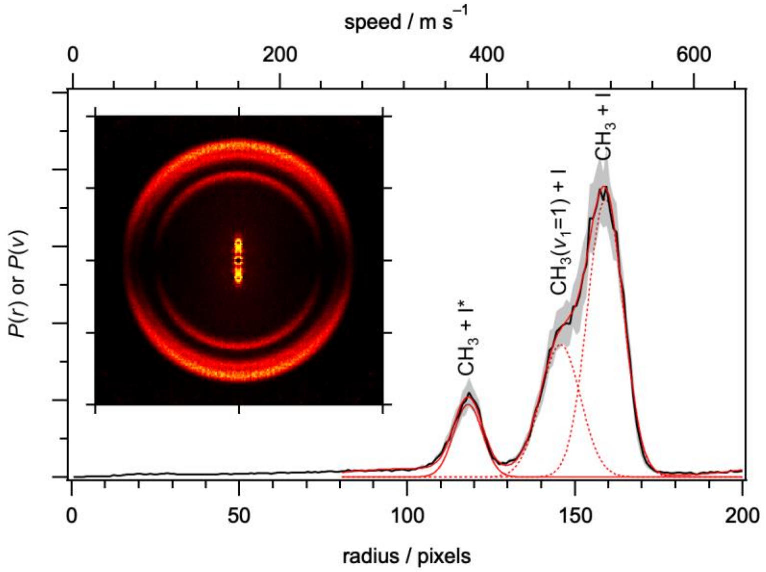

Figure S1 Velocity-map ion image and normalized radial (speed) distribution for I and I* products formed from the photolysis of CH_3I at 280 nm. Shaded areas represent 1σ uncertainties from repeated measurements. Dashed and solid red lines are the fitted individual Gaussian components and overall fit, respectively.

Fragment time profiles

Time profiles for the heights of the m/z = 15, 42, 43, 58, 85, and 100 peaks in the mass spectra as a function of the time delay between the UV and VUV beams (Δt – t_{UV} – t_{VUV}) are shown in Figure S2. The time profiles show two-color contributions after subtraction of VUV only signals i.e. [UV + VUV] – [VUV].

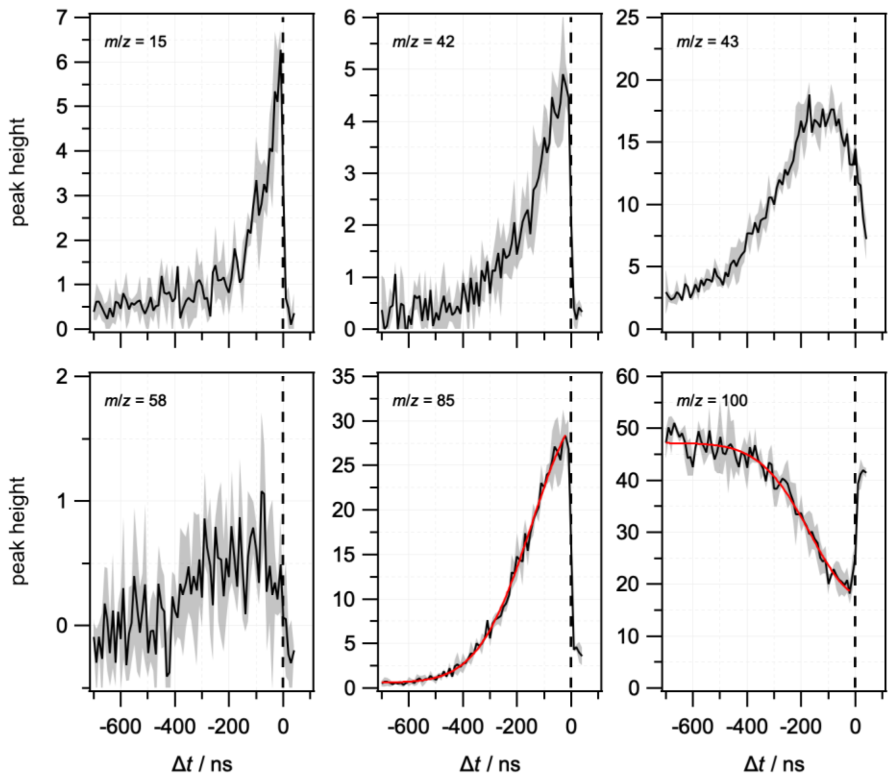

Figure S2 Time-of-flight mass spectra peak heights as function of Δt (delay between UV and VUV laser pulses) for m/z = 15, 42, 43, 58, 8, and 100 ions. Values of $\Delta t < 0$ correspond to the UV beam preceding the VUV beam. The measurements used a UV pulse energy of 150 μJ (fluence 19 mJ cm^{-2}). Shaded areas represent 1σ uncertainties derived from repeated measurements.

Photolysis power dependence

Ion images of the m/z = 15, 43, 58, and 85 photofragments resulting from excitation of AcAc at 280 nm were acquired using different pulse energies to test for effects of multiphoton excitation. The speed distributions $P(v)$ obtained using UV pulse energies that are a factor of 10 different are shown in Figure S3 below. The speed distributions are identical within measurement uncertainties. Power dependences were also explored using time-of-flight mass spectra acquired with UV pulse energies spanning the range 40–420 μJ (fluence 5–54 mJ cm^{-2}). The double logarithmic plots of peak height versus pulse energy are showin in Figure S4 and the gradients are compiled in Table S1.

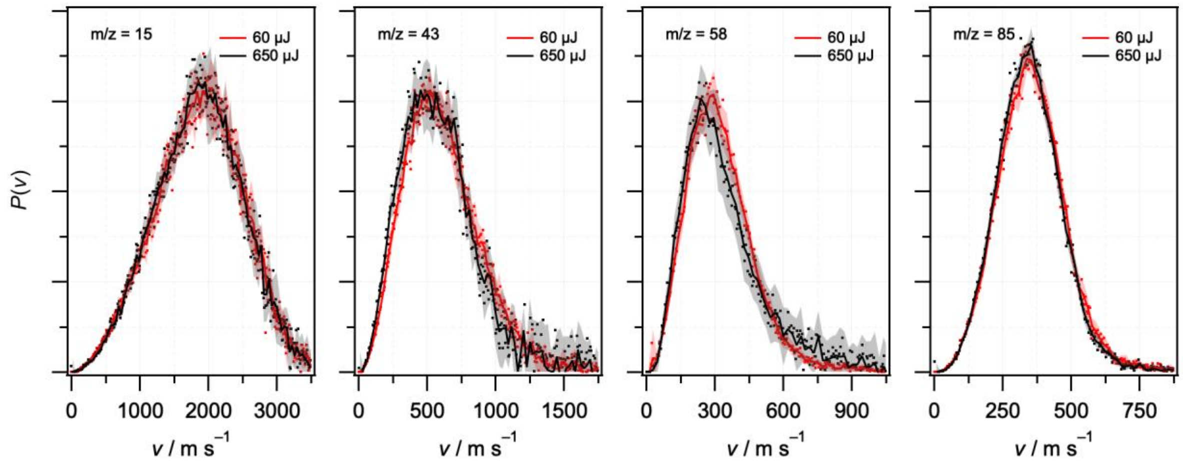

Figure S3 Speed distributions $P(v)$ for m/z = 15, 43, 58, and 85 photofragments following excitation of AcAc at 280 nm using pulse energies of 60 μJ and 650 μJ (fluences of 8 mJ cm^{-2} and 83 mJ cm^{-2}, respectively). Shaded areas represent 1σ uncertainties derived from repeated measurements.

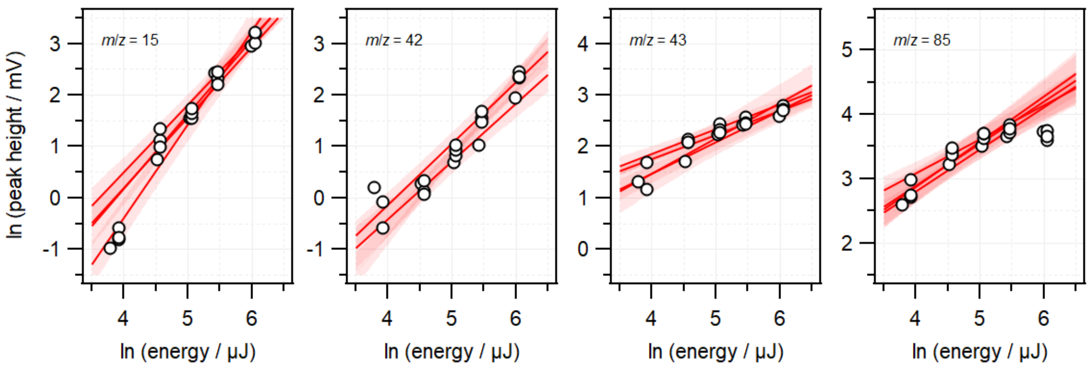

Figure S4 Double logarithmic plots of peak height versus UV pulse energy. Red lines are linear fits, with shaded areas representing 1σ prediction bands. The gradients are compiled in Table S1

Table S1 Gradients of the log-log plots of peak height versus UV pulse energy derived from time-of-flight mass spectra shown in Figure S4. Uncertainties are 1σ.

m/z	n
15	1.49±0.23
42	1.35±0.23
43	0.56±0.10
85	0.74±0.10

Fragment momentum distributions

The momentum distributions $P(p)$ for the m/z = 42 and 58 fragments are shown in Figure S5.

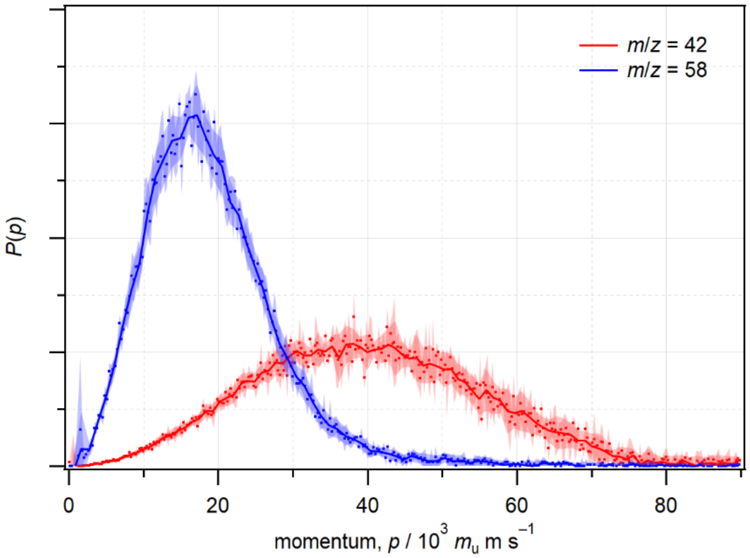

Figure S5 Normalized momentum distributions $P(p)$ for m/z = 42 and m/z =58 fragments following excitation of AcAc at 280 nm. Shaded areas represent 1σ uncertainties derived from repeated measurements.

Ab initio calculations

Ab initio calculations were performed at the B3LYP/cc-pVDZ level of theory using the GAMESS package (version 2020 R2).[7] Optimized geometries and harmonic frequencies of the enolone and diketone tautomers, the tautomerization transitions state TS1, and the molecular dissociation transitions states TS2–TS8 were calculated. The TS labels are the same as those used for the equivalent stationary points characterized at the CBS-QB3 level by Antonov et al.[8] Cartesian coordinates are listed in Table S2 and the electronic energies and harmonic frequencies are compiled in Table S3.

Table S2 Cartesian coordinates for transition states TS1–TS8. The vectors indicate the displacements associated with the imaginary mode.

TS1	C 1.7496396192 1.2415575386 0.4017300782<br>C 1.2843796578 -0.1352796349 0.1115553394<br>C -0.0454178656 -0.7110149013 0.2272625254<br>C -1.2651360016 0.0635217463 -0.0405721008<br>C -2.5878660338 -0.6390367164 0.2253836229<br>O 2.0583798806 -1.0290011053 -0.3721806968<br>O -1.2390403407 1.2099779609 -0.4848333865<br>H 1.0674357427 1.9204103143 -0.1388803246<br>H 1.6133808718 1.4639957022 1.4734917409<br>H 2.7941860069 1.3921935392 0.1010282393<br>H 0.9249739881 -1.6003308082 -0.5008213998<br>H -0.0992297594 -1.4616745996 1.0300188583<br>H -2.7495921537 -0.7220428594 1.3151638787<br>H -3.4091401609 -0.0562080303 -0.2126914180<br>H -2.5887318802 -1.6629905247 -0.1825516666	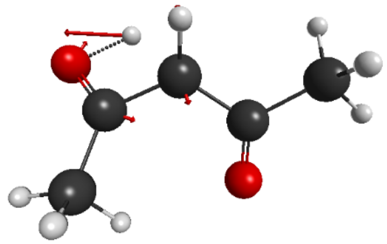
TS2	C -2.6437426359 0.5603985132 0.0065415578<br>C -1.2316728190 0.0436238307 -0.0010484219<br>C -0.1444671412 0.9389566156 -0.0052167105<br>C 1.1849364650 0.5439066420 -0.0133748837<br>C 1.5208227748 -1.5396256275 0.0094196111<br>O -1.0681276224 -1.2178147223 -0.0057373986<br>O 2.2870199707 0.9629095321 -0.0250532386<br>H -2.6868511556 1.6576314658 0.0484350747<br>H -3.1629400643 0.2063439462 -0.8993340253<br>H -3.1799275406 0.1346655064 0.8701593962<br>H 0.1986399989 -1.3052492127 -0.0044579850<br>H -0.3092937062 2.0168314497 -0.0039428772<br>H 2.1393774784 -1.4375706253 -0.8911448389<br>H 2.1315395564 -1.4093421191 0.9117130244<br>H 1.1427495892 -2.5867768638 0.0256018727	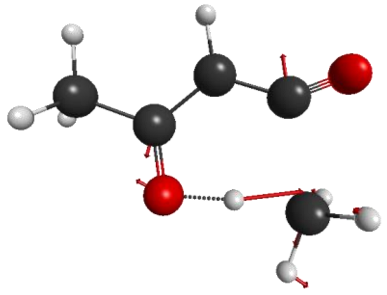

TS3	C 1.0853372979 1.1842569748 0.6879495115<br>C 1.2414832799 -0.1042999253 0.1460291379<br>C 0.1346758663 -0.9610794818 0.0258257417<br>C -1.1835147500 -0.5160927578 0.0412278395<br>C -1.4190062093 1.5572319650 -0.5474736530<br>O 2.4351516869 -0.4385584153 -0.3946290515<br>O -2.3044930975 -0.8512820860 0.1879580543<br>H 0.6046485707 1.2643987064 1.6669778772<br>H 1.8951970155 1.8981973676 0.5083522111<br>H -0.1613317161 1.3607158564 0.0076156262<br>H 2.3146354742 -1.2089154243 -0.9754024963<br>H 0.2396134021 -2.0190412797 -0.2314978890<br>H -1.9112094045 1.1006896136 -1.4171213704<br>H -2.1639918571 1.7665820768 0.2326145823<br>H -0.9930174076 2.5196571828 -0.8951691030	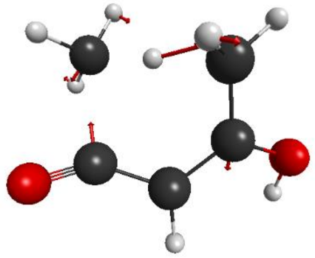
TS4	C -1.8206644634 1.3412359958 -0.6026989733<br>C -1.1325428736 0.1417819228 -0.0698948766<br>C -0.0100415696 -0.4614763576 0.2090246726<br>C 1.3863857750 -0.0213955525 0.1344508962<br>C 2.1988990234 -0.6033211128 -1.0131995783<br>O -2.2106062464 -1.0460032704 0.3101901880<br>O 1.8911997328 0.7089492656 0.9756616376<br>H -1.0560707051 2.0887235938 -0.8645727280<br>H -2.4142404459 1.1102096980 -1.5025354384<br>H -2.4959016490 1.7976048821 0.1416559723<br>H -2.6116534617 -0.8245980169 1.1742584954<br>H -1.1624005578 -1.4691873078 0.5129949722<br>H 3.2183495358 -0.1932275533 -0.9853988053<br>H 2.2377546837 -1.7024937349 -0.9276495442<br>H 1.7242415961 -0.3743063866 -1.9826331384	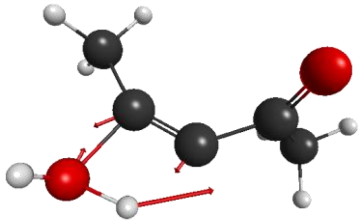
TS5	C -2.5310616721 0.7539398859 -0.2316306445<br>C -1.2024053349 0.4008713176 0.0527873597<br>C 0.0684146304 0.8440116492 0.0820851932<br>C 1.3736562602 0.1582999797 -0.0298766357<br>C 1.4610339155 -1.3437093486 -0.2556877279<br>O -1.4140260155 -1.2252600871 0.1795291446<br>O 2.3965089648 0.8257860160 0.0585735298<br>H -3.2579568257 0.6577395601 0.5887907566<br>H -2.7021572963 1.6111784480 -0.8897987245<br>H -2.3771970102 -0.8554514225 -0.2823862324<br>H -1.5005857072 -1.4884786592 1.1190262707<br>H 0.1656573125 1.9303993021 0.1751587773<br>H 0.7964962191 -1.6790136040 -1.0660995733<br>H 2.5054372496 -1.5899730975 -0.4889379141<br>H 1.1641953485 -1.8929559928 0.6532796940	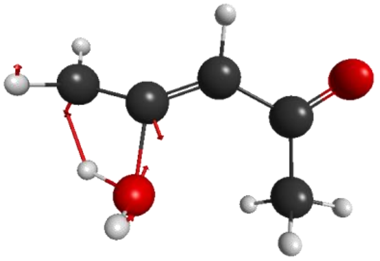
TS6	C -1.3255414353 1.3155841477 -0.4797360743<br>C -1.2049745302 -0.0944863863 -0.0193801446<br>C -0.0003757531 -0.7608887666 0.1490857751<br>C 1.3950675348 -0.0169272147 0.1952618604<br>C 2.2864904851 -0.9134967720 -0.4821236221<br>O -2.3943063808 -0.6899375738 0.1933371010<br>O 1.5154733081 1.1710906582 0.4582164837<br>H -0.5099988967 1.9055466640 -0.0305146465<br>H -1.2002759483 1.3748814837 -1.5761843807<br>H -2.3154147515 1.7182260375 -0.2234915739<br>H -2.2480595798 -1.5997739836 0.5039341983<br>H -0.0414947766 -1.7337537615 0.6608003191<br>H 3.2765069340 -0.5621781415 -0.7943514917<br>H 2.2089158870 -1.9786053142 -0.2382144651<br>H 0.6953112025 -1.0260930902 -0.9532382652	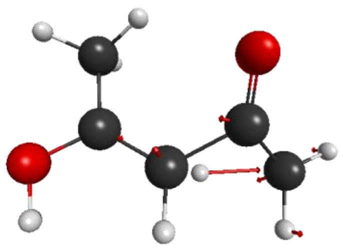

TS7	C 2.2488853034 0.7900433239 0.3094238124<br>C 1.1598224059 -0.0972616765 -0.2073991792<br>C 0.2051997646 0.3230403145 -1.1428987286<br>C -1.4177026584 0.2052660612 -0.0143445663<br>C -1.6864540022 -1.1519709552 0.0197946375<br>O 1.0564253596 -1.2542769272 0.4024977468<br>O -1.6934291098 1.2594230537 0.4704538063<br>H 2.6286644042 1.4715189942 -0.4635552351<br>H 1.8352272319 1.4011009847 1.1334009837<br>H 3.0664286602 0.1839152197 0.7244959167<br>H -0.1958694259 -0.4088224855 -1.8453130142<br>H 0.3169748825 1.3330183891 -1.5439006430<br>H -2.4955900549 -1.4530675685 0.6929471948<br>H -1.5298508294 -1.7509489855 -0.8774756981<br>H 0.0497521782 -1.5109079166 0.3486020822	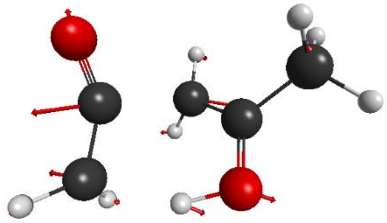
TS8	C 1.8706247026 1.1890395071 -0.1925978695<br>C 1.3282818828 -0.2192230044 -0.0618216065<br>C 0.1345022120 -0.5769528375 -0.9014717510<br>C -1.5508818940 0.2280250475 -0.0231656304<br>C -2.3080989809 -0.9371829726 0.0984798515<br>O 1.8055601735 -1.0330545812 0.7124283505<br>O -1.3434177036 1.3056645235 0.4195744246<br>H 2.2283016120 1.3653025947 -1.2219715466<br>H 1.0635047450 1.9159144481 -0.0008084057<br>H 2.6947542854 1.3401814390 0.5174675522<br>H -0.0190783796 0.0578868104 -1.7851014208<br>H 0.1798095547 -1.6358600505 -1.2118078668<br>H -3.1916637951 -0.9141780737 0.7517579670<br>H -2.3379384830 -1.6041626964 -0.7640271602<br>H -0.8539594626 -1.0971449768 0.0806517421	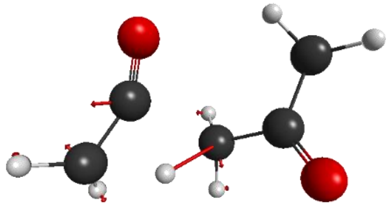

Table S3 Electronic energies (first row, +345 Hartree) and harmonic vibrational frequencies (cm^{-1}) for the enolone and diketone tautomers of AcAc, and the molecular dissoc7iation transition states used in the RRKM calculations described in the main text.

enolone	diketone	TS1	TS2	TS3	TS4	TS5	TS6	TS7	TS8
−0.62486	−0.61420	−0.52670	−0.53147	−0.48032	−0.47510	−0.47013	0.48633	-0.53155	−0.47644
88.1	54.7	1936.6*i*	1373.1*i*	1518.9*i*	1397.6*i*	1392.0*i*	1360.2*i*	409.7*i*	1762.7*i*
119.3	66.9	85.6	40.2	107.5	53.7	59.9	77.2	84.0	43.9
157.7	135.5	101.5	69.3	149.9	109.1	145.4	154.5	130.5	75.6
188.2	163.1	126.3	152.9	199.4	130.7	177.6	174.6	159.7	139.6
233.8	164.2	166.2	209.0	302.7	171.7	200.8	228.2	223.3	172.5
378.9	323.9	185.9	285.3	329.6	237.6	282.9	365.3	337.0	193.2
396.2	417.4	309.5	367.9	388.0	325.5	341.7	388.6	416.5	335.4
511.8	487.9	411.3	382.7	468.4	336.8	364.9	473.0	447.2	403.3
557.0	504.7	451.0	495.1	479.2	399.7	411.6	497.8	501.0	429.1
649.5	546.2	554.6	517.4	506.3	491.9	488.6	541.5	546.5	519.9
649.6	623.0	590.8	595.9	553.5	494.5	561.9	592.7	594.7	547.4
778.3	790.8	640.6	621.9	575.9	587.8	567.6	625.4	632.8	615.5
921.5	803.6	693.1	639.4	584.1	630.9	589.0	672.2	687.0	662.0
944.8	894.3	826.6	664.0	630.3	651.2	756.0	766.0	784.7	741.2
1003.6	933.6	944.2	714.1	683.7	774.5	795.7	819.2	842.6	821.8
1019.9	990.0	984.5	838.6	792.8	945.8	811.4	857.3	917.2	849.9
1025.0	1054.5	1003.8	977.7	813.4	955.8	836.1	901.8	950.1	943.2
1033.4	1067.9	1029.0	1008.9	924.6	1024.9	921.2	1016.1	994.3	968.9
1049.0	1131.5	1048.2	1040.4	980.8	1048.1	997.9	1030.4	1027.6	1051.4
1187.3	1181.8	1151.5	1167.5	1023.8	1055.1	1026.5	1035.6	1039.1	1064.3
1283.6	1245.2	1177.6	1202.9	1135.8	1068.3	1032.4	1151.6	1060.8	1086.3
1366.6	1261.6	1296.8	1226.2	1203.2	1264.4	1125.9	1169.7	1103.7	1120.2
1376.7	1367.3	1350.8	1332.2	1211.9	1351.4	1240.5	1210.4	1315.1	1240.7
1398.1	1368.3	1372.3	1364.1	1290.5	1356.7	1274.7	1291.7	1374.5	1245.9
1433.0	1431.3	1420.4	1374.8	1308.9	1389.3	1370.4	1363.6	1409.9	1364.9
1442.2	1434.4	1422.3	1422.2	1401.8	1432.4	1385.6	1415.3	1422.9	1419.4
1447.5	1441.4	1430.8	1435.3	1408.8	1438.4	1428.3	1424.2	1451.7	1436.8
1448.0	1441.8	1445.9	1437.3	1440.4	1440.4	1429.5	1443.1	1466.3	1446.3
1494.5	1454.2	1454.0	1519.1	1517.8	1454.3	1451.5	1468.7	1478.6	1450.1
1664.9	1787.4	1554.5	1621.0	1600.2	1743.6	1731.6	1597.8	1595.4	1681.6
1689.2	1813.5	1731.6	1714.8	1617.1	1843.2	1760.6	1655.8	1933.5	1771.9
2832.5	3027.8	1904.8	1998.4	2001.0	1877.1	1888.1	1774.6	2505.8	1990.0
3032.9	3028.1	3025.2	2958.3	2993.0	3022.0	3040.8	3019.7	3027.6	3027.7
3037.2	3069.9	3033.3	3039.3	3099.2	3027.1	3074.1	3083.6	3110.4	3041.6
3098.4	3095.6	3094.1	3108.7	3123.0	3085.2	3112.0	3095.0	3131.9	3097.9
3099.3	3095.8	3097.8	3109.0	3159.2	3096.4	3151.5	3110.0	3145.8	3098.6
3146.2	3142.1	3099.6	3141.4	3171.8	3122.8	3167.4	3142.1	3158.1	3120.4
3150.7	3152.3	3146.6	3175.9	3209.9	3141.6	3189.1	3205.0	3231.3	3148.6
3211.6	3152.4	3157.9	3207.2	3718.4	3655.9	3635.0	3724.0	3240.3	3233.2

References

(1) Chandler, D. W.; Houston, P. L. Two-Dimensional Imaging of State-Selected Photodissociation Products Detected by Multiphoton Ionization. *J. Chem. Phys.* **1987**, *87* (2), 1445–1447.

(2) Eppink, A. T. J. B.; Parker, D. H. Methyl Iodide A-Band Decomposition Study by Photofragment Velocity Imaging. *J. Chem. Phys.* **1998**, *109* (12), 4758–4767. https://doi.org/10.1063/1.477087.

(3) Eppink, A. T. J. B.; Parker, D. H. Energy Partitioning Following Photodissociation of Methyl Iodide in the A Band: A Velocity Mapping Study. *J. Chem. Phys.* **1999**, *110* (2), 832–844. https://doi.org/10.1063/1.478051.

(4) Gardiner, S. H.; Lipciuc, M. L.; Karsili, T. N. V.; Ashfold, M. N. R.; Vallance, C. Dynamics of the A-Band Ultraviolet Photodissociation of Methyl Iodide and Ethyl Iodide via Velocity-Map Imaging with 'Universal' Detection. *Phys. Chem. Chem. Phys.* **2015**, *17* (6), 4096–4106. https://doi.org/10.1039/C4CP04654D.

(5) Zhu, Q.; Cao, J. R.; Wen, Y.; Zhang, J.; Zhong, X.; Huang, Y.; Fang, W.; Wu, X. Photodissociation Channels and Energy Partitioning in the Photofragmentation of Alkyl Iodides. *Chem. Phys. Lett.* **1988**, *144* (5), 486–492. https://doi.org/10.1016/0009-2614(88)87301-9.

(6) Fan, H.; Pratt, S. T. Determination of Spin−Orbit Branching Fractions in the Photodissociation of Halogenated Hydrocarbons. *J. Phys. Chem. A* **2007**, *111* (19), 3901–3906. https://doi.org/10.1021/jp0670034.

(7) Barca, G. M. J.; Bertoni, C.; Carrington, L.; Datta, D.; De Silva, N.; Deustua, J. E.; Fedorov, D. G.; Gour, J. R.; Gunina, A. O.; Guidez, E.; et al. Recent Developments in the General Atomic and Molecular Electronic Structure System. *J. Chem. Phys.* **2020**, *152* (15), 154102. https://doi.org/10.1063/5.0005188.

(8) Antonov, I.; Voronova, K.; Chen, M.-W.; Sztáray, B.; Hemberger, P.; Bodi, A.; Osborn, D. L.; Sheps, L. To Boldly Look Where No One Has Looked Before: Identifying the Primary Photoproducts of Acetylacetone. *J. Phys. Chem. A* **2019**, *123* (26), 5472–5490. https://doi.org/10.1021/acs.jpca.9b04640.
